# MIEO: a micro-invasive endoscopic operation port system for transluminal interventions—an acute and survival porcine study

**DOI:** 10.1007/s00464-020-07518-3

**Published:** 2020-04-06

**Authors:** D. Wilhelm, T. Vogel, A. Jell, S. Brunner, M. Kranzfelder, N. Wantia, H. Feussner, D. Ostler, S. Koller

**Affiliations:** 1 Technische Universität München, Fakultät für Medizin, Klinik Und Poliklinik für Chirurgie, Klinikum Rechts Der Isar, Ismaningerstr. 22, München, 81675 Germany; 2 Arbeitsgruppe für Minimal-Invasive Technologie Und Intervention, Klinikum Rechts Der Isar, München, Germany; 3 Technische Universität München, Institut für Virologie Und Bakteriologie, Klinikum Rechts Der Isar, München, Germany; 4 Röchling Medical Waldachtal AG, Waldachtal, Deutschland, Germany

**Keywords:** Overtube, NOTES, Transluminal surgery, Infection, Sterilization, Sealing

## Abstract

**Background:**

A reliable and sterile access through the intestinal wall to ease flexible endoscopic transluminal interventions is still appealing but lacks a suitable port system.

**Methods:**

In a granted industry cooperation, we developed the MIEO-Port, a flexible three components overtube system that provides a temporary hermetic sealing of the intestinal wall to allow endoscopic disinfection and manipulation to gain access to the abdominal cavity. The port features an innovative head part which allows for coupling the port to the intestinal wall by vacuum suction and for controlled jetting the isolated intestinal surface with a disinfectant. The device was tested in vivo in 6 pigs for acute and long-term usability. All animal tests were approved by the local ethics committee.

**Results:**

In the acute experiment, the port system supported sealed endoscopic mucosa resection and transluminal cholecystectomy. In the survival study on 5 animals, the MIEO-Port proved its reliability after transcolonic peritoneoscopy. In one animal, a port dislocation occurred after extensive retroperitoneal preparation, one animal revealed bacterial contamination at necropsy; however, all animals showed a favourable course over ten days and offered no signs of peritonitis or abscedation during post-mortem examination.

**Discussion:**

To the best of our knowledge, the MIEO-Port system is the first device to provide a reliable and sterile flexible access to the peritoneal cavity that can be used throughout the entire gastrointestinal tract regardless of the access route and which combines hermetic sealing with local sterilization. Further studies are warranted.

**Electronic supplementary material:**

The online version of this article (10.1007/s00464-020-07518-3) contains supplementary material, which is available to authorized users.

## Background

Although the hype on transluminal procedures has faded in recent years, the idea is still charming and many researchers worldwide continue to develop innovative concepts that will one day allow for less invasive, or better, less stressful interventions. Nowadays, researchers are no longer focusing on replacing conventional laparoscopic interventions but on developing new principles that offer real advantages to the current standard. In this regard, peroral endoscopic myotomy [1] and transanal total mesorectal excision [2] deserve notion as good examples to highlight the potential of transluminal surgery. Despite any future application, a safe and reliable technique to penetrate the natural orifice still represents one of the main obstacles and requires, particularly in the colon, technical precautions to prevent infection. Since this goal is difficult to achieve in a highly dynamic environment with continuous peristalsis and intestinal fluids passing, primary focus in past years was given to occlusion devices that block the entire intestinal lumen. Linke et al. in 2013 proposed a balloon occluder to be placed cephalad to the penetration site [3, 4] and which was tested against conventional laparoscopic cholecystectomy [5]. A similar technology was proposed by Du et al. [6]. The technique to enter the abdomen, however, has remained almost unchanged. While some groups chose direct endoscope penetration [7, 8], our group was the first to propose usage of a transluminal overtube [9] to penetrate the intestinal wall and establish a permanent gate to the abdominal wall. The so-called ISSA (innovative safe and sterile access) worked similarly to a laparoscopic trocar and to a certain extent was able to separate the endoscope from the intestinal lumen. However, moving the overtube in and out the peritoneal cavity always caused spillage of intestinal fluids into the abdomen. That is, why maybe Senft combined an identical overtube system with an intestinal occluder [3]. Also effective disinfection of the entry site deems essential to minimize the risk of contamination but is confined to simply washing out the intestine [10–12]. This might be acceptable in the rectum where disinfecting solutions can directly be applied, but fails in the colon. Accordingly, peritoneal contamination is observed as a constant side effect of transcolonic surgery [13].

In summary, although certain goals for a universal transluminal access system have been achieved, key aspects such as a proper sealing of the entry site and improved disinfection are still unmet. With the MIEO, we aimed to develop a universal port system that can be used anywhere throughout the gastrointestinal tract and for any transluminal application, and that combines full flexibility for endoscopic manipulation with high level of sterility maintenance and safety. In this manuscript, we focus on the in vivo evaluation of the port system, while technical details and ex vivo studies will be published separately, as well as we did not intend to develop a new transluminal procedure.

## Methods

### MIEO-Port

**Fig. 1:**
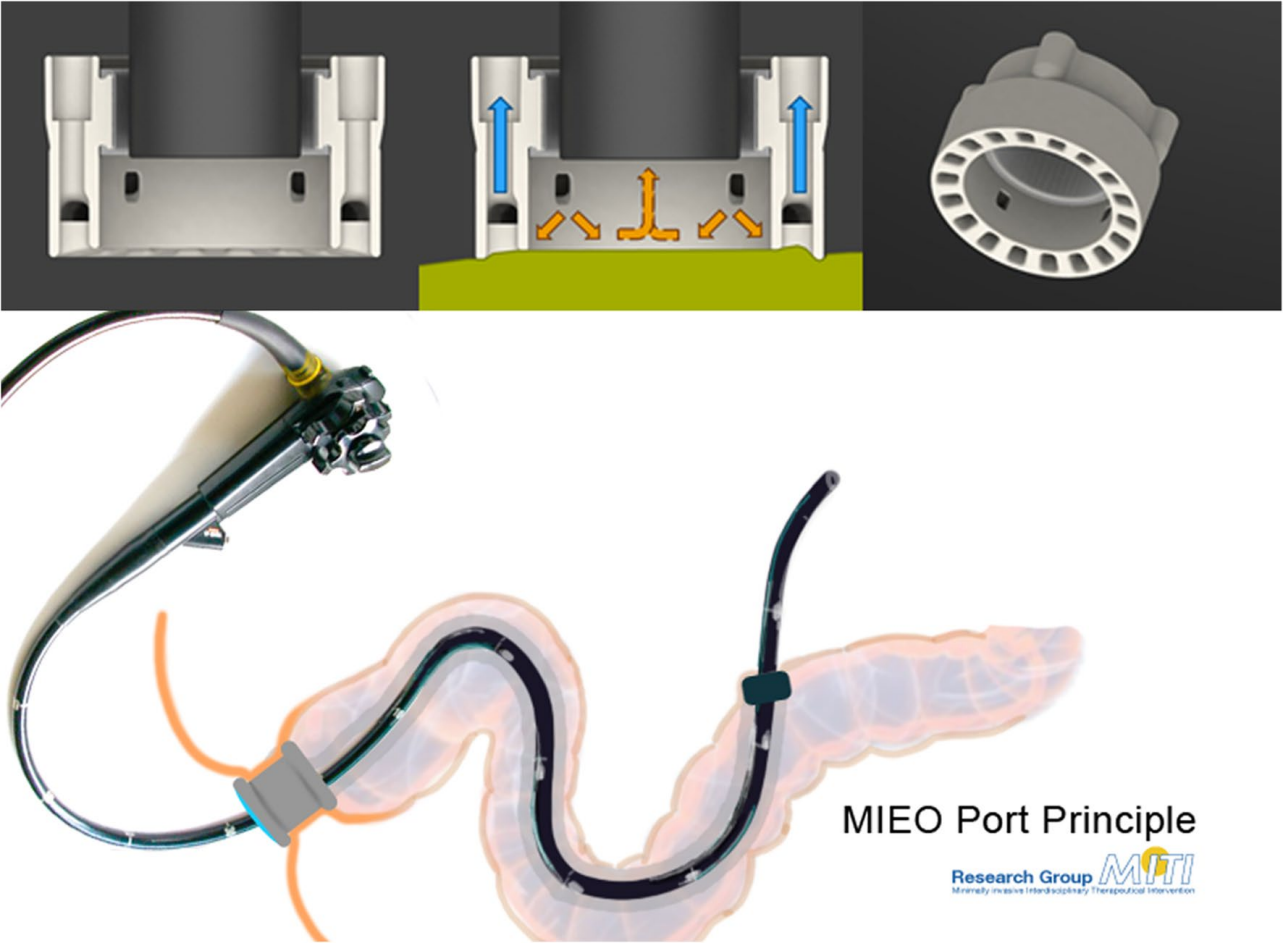
The head part provides multiple openings that with application of negative pressure allows for docking the MIEO to the intestinal wall (blue arrows). Several jets at the inner surface can be flushed with a disinfection solution and subsequently can sterilize the intestinal surface (orange arrows). The disinfection solution is drained via the working channel of the scope. The entire MIEO-Port consists of the head component (left side), the flexible hose and the outer cap with connectors to facilitate the port functionality (Color figure online)

The MIEO consists of three main components. Core component is the 20-mm-wide head piece which combines all functionalities necessary for the reliable and sterile transluminal access. It offers 18 suction slots which are circularly arranged at its end plane and which can be evacuated via two independent suction channels to attach the MIEO-Port to the intestinal wall. With the endoscope held internally of the head section by means of an inflatable ring-balloon, a small chamber is created that can be flushed with a disinfectant to sterilize the isolated intestinal segment. To improve the disinfection process, 4 spray nozzles facilitate high-pressure irrigation. The disinfection agent is drained via the endoscope working channel that also provides insufflation of the intubated organ, visual control and navigation abilities while manoeuvring the port system. The head section with its supply lines is connected via a flexible plastic hose of individual length to an outer cap which uses several Luerlock connectors to link the supply lines to conventional laparoscopic devices. Stabilization of the port inside the anus or mouth is another cap feature as well as it offers pressure controlled insufflation of the intestine or abdominal cavity (Fig. 1).

The head piece and outer cap of the MIEO-prototype are computer designed and fabricated by 3D printing technology. Defined openings in the head component and outer cap serve for insertion of the supply lines. The outer connectors have a standard Luerlock shape to be used with conventional material. The head part and outer cap are linked via a flexible plastic hose, which contains the supply lines and which can be shortened to the required length depending on the designated entry site. The inner ring-balloon for fixation of the endoscope is made of silicone by vacuum casting. The outer sealing hood is sputtered of silicon in a negative profile to match the cap geometry, the system is sterilisable.

### Procedure

**Fig. 2:**
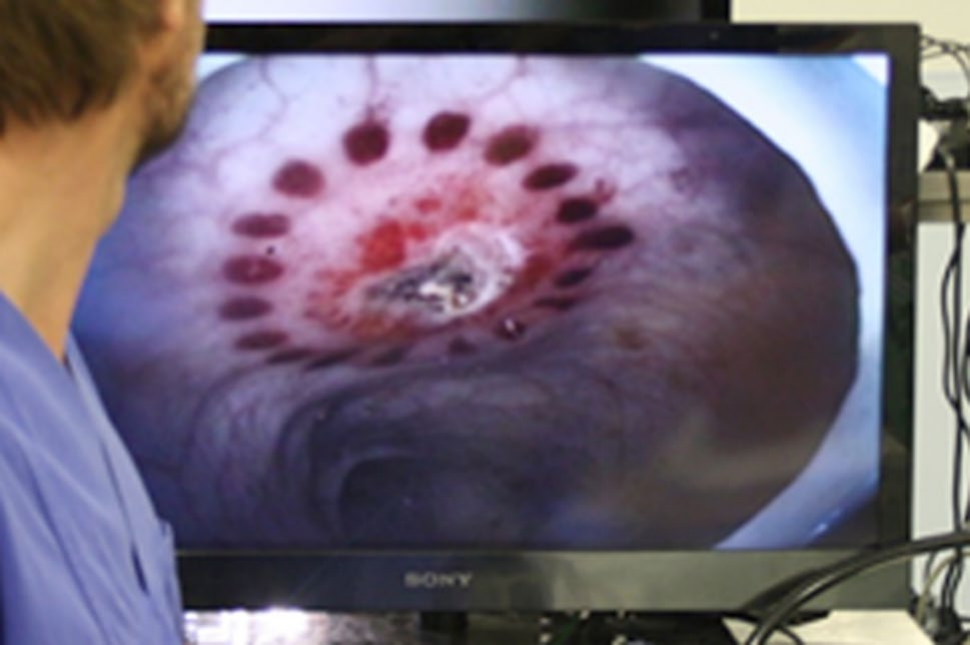
Small punctiform haemorrhages surrounding the entry site, which are caused by the docking the port to the intestinal wall, can be used for localization and closure of the access point

The MIEO-Port procedure starts with insertion of a standard flexible endoscope (diameter 8–13 mm) into the overtube and its fixation inside the head component by insufflating the internal balloon. With the endoscope firmly anchored to the head section the entire system is intubated into anus or mouth and then advanced to the designated entry site. After intubation of the port system, the outer cap is placed into the orifice to prevent from dislocation and to allow for air tight sealing. With reaching the access site after conventional endoscopic manipulation, the head of MIEO is couched to the intestinal wall and subsequently docked by vacuum suction. Now the sterilization process is initiated by flushing the disinfectant on the isolated intestinal surface. Disinfection is visually controlled via the endoscope. After evacuating the fluid via the working channel, the endoscope is released from the port by venting the fixation balloon. By endoscopic manipulation (e.g. needle knife incision), the intestinal wall is subsequently perforated and the endoscope advanced through the intestinal wall into the peritoneal cavity for flexible endoscopic surgery. Throughout the entire procedure, the port system remains firmly docked to the intestinal wall sealing both the penetration site and inserted endoscope from secondary contamination. After completion of the procedure, the endoscope is withdrawn into the intestinal lumen and the port undocked from the fixation site by stopping vacuum suction. Closure of the entry site is realized with Over-the-scope-clip (Ovesco Endoscopy AG, Tübingen, Germany) application. Punctual small mucosal haemorrhages (Fig. 2) that result from suction at the docking site encircle the penetration site thus facilitating relocation and clip placement.

For driving the port system functional we used two conventional pressure controlled suction devices set to 600 mmHg (80 kPa) negative pressure and a laparoscopic insufflator (Endoflator, Karl–Storz, Tuttlingen, Germany). Application of the disinfection solution was realized by hand with a 20 ml syringe. In prior ex vivo tests, the functionality and disinfection ability of the port cap had been evaluated in comparison to conventional methods and other disinfectants. During these tests, the MIEO-Port with application of Octenisept® (Schülke & Mayr, Norderstedt, Germany) showed superiority to any other method (e.g. standard rinsing, application of water or povidone-iodine, etc.) and reached a complete or near to complete elimination of the intestinal bacteria flora. Accordingly, this setup best disinfection functionality for was used in process of the in vivo studies. As the results of the ex vivo evaluation will be published separately, they are not included in this manuscript.

The principle of the MIEO-Port and its application is further displayed in the supplemented video.

### Evaluation study

The MIEO was evaluated in an acute and survival animal study. The acute model served for proof of concept of the MIEO-Port by performing a sealed mucosal resection and a transluminal hybrid NOTES cholecystectomy. Both procedures were chosen for feasibility and as the focus was given to the transluminal access technique and not the procedure itself. For mucosal resection the MIEO-Port and standard flexible instruments (MTW polypectomy snare 99,052,011,212 and biopsy forceps 99,060,502,805, MTW Endoskopie, Wesel, Germany) were used, while the cholecystectomy was completed with an endoscopic hook knife (Olympus HookKnife®, Olympus Deutschland GmbH, Hamburg, Germany) after injection with an endoscopic injection needle (Injection needle 0,910,718,212, MTW Endoskopie) and by laparoscopic support for exposure (atraumatic laparoscopic grasper, Karl Storz, Tuttlingen, Germany). Closure of the cystic artery and duct were achieved by endo-clip application (ResolutionClip™, Boston Scientific Medizintechnik GmbH, Ratingen, Germany).

In the survival study, a transluminal flexible peritoneoscopy was performed after gaining access to the peritoneal cavity according to the technique described. Before starting the procedure, the lower parts of the colon were cleansed by tap water enemas. For the acute and survival experiment, the animals were anaesthetized by an approved veterinarian and according to the study protocol. However, in the acute experiment, the pig was euthanized with completion of the operation, in the survival model animals were kept alive for follow-up observation. Animals were allowed to eat and drink the day of operation. Euthanasia in the survival group was postponed to the 10th postoperative day. During the survival period all animals were evaluated twice a day for adverse events. During post-mortem examination, animals were evaluated for signs of infection or any other complication as well as microbiological swabs of the abdomen and nearby the entry site were taken. The entry site was explored for complication-free healing and the docking site of the port was checked for necrosis or other procedure related complications. During the local evaluation special attention was given to the tiny haemorrhages observed as the result of vacuum suction (see below), which were carefully inspected from the in- and outside.

We performed no statistical analyses due to the non-comparative study design, descriptive values and tables were calculated using Microsoft Excel, Office 2016 Pro® for Windows.

All procedures were performed by the same experienced endoscopist (D. W.), approval of the study protocol was obtained from the local ethics committee (ROB-55.2–2532.Vet_02-18–172; Regierung von Oberbayern) prior to experiments. As requested by the ethical commitee the number of animals was limited to the indicated cohort. No other IRB approval or informed consent deemed necessary.

## Results

### Sealed endoscopic mucosal resection

**Fig. 3:**
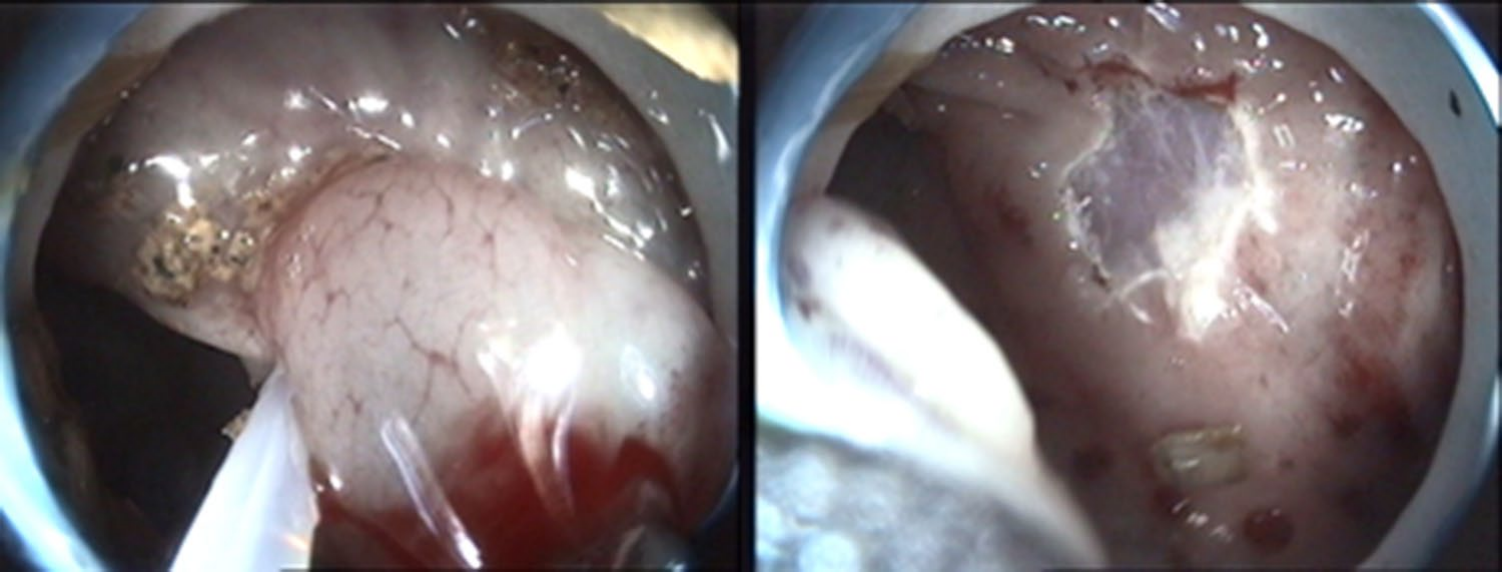
Endoscopic findings during sealed endoscopic mucosal resection. On the left side one can identify a grasper that supported exposure of the mucosa that was then dissected by hot snare resection

In the acute model, the port system was tested in concept on a 63 kg female landrace pig for sealed endoscopic mucosal resection. As with healthy porcines no intestinal pathology was present, we intended to just resect a small part of the mucosa. For this purpose, the port system was coupled to the intestinal wall, fixed by suction and the resection site disinfected. With the endoscope uncoupled  from the MIEO-Port an endoscopic snare loop was deployed but failed to grasp the mucosa due to the absence of a pathology and due to the fact, that the MIEO-Port spanned plane the intestine after docking. As injecting saline into the submucosa for lifting the mucosa did not help either, we changed to a two channel endoscope (KST 13806P, Karl–Storz, Tuttlingen, Germany) that allowed for lifting the mucosa by use of an additional grasper and for subsequent resection with the deployed snare loop (Fig. 3). The procedure was repeated several times within the same animal and worked identically well but for one site causing an intestinal perforation due to full thickness resection. However, even in this situation the MIEO-Port remained stable in position and reliably sealed the interventions site.

### Transluminal cholecystectomy

The transluminal endoscopic cholecystectomy was performed according to a previously published technique [14]. Again, the port system was coupled to the colon in a height of 30 cm from the anal verge followed by disinfection of the entry site. The intestine was subsequently cut by use of an endoscopic knife and this incision then dilated by an inflatable balloon (CRE® balloon dilator, 08,714,729,202,004, Boston Scientific Medizintechnik GmbH, Ratingen, Germany). The endoscope was advanced into the peritoneal cavity and easily manoeuvred to the liver. For documentary reasons the procedure was observed by conventional laparoscopy (30° laparoscopic endoscope, Karl–Storz, Tuttlingen, Germany).

**Fig. 4:**
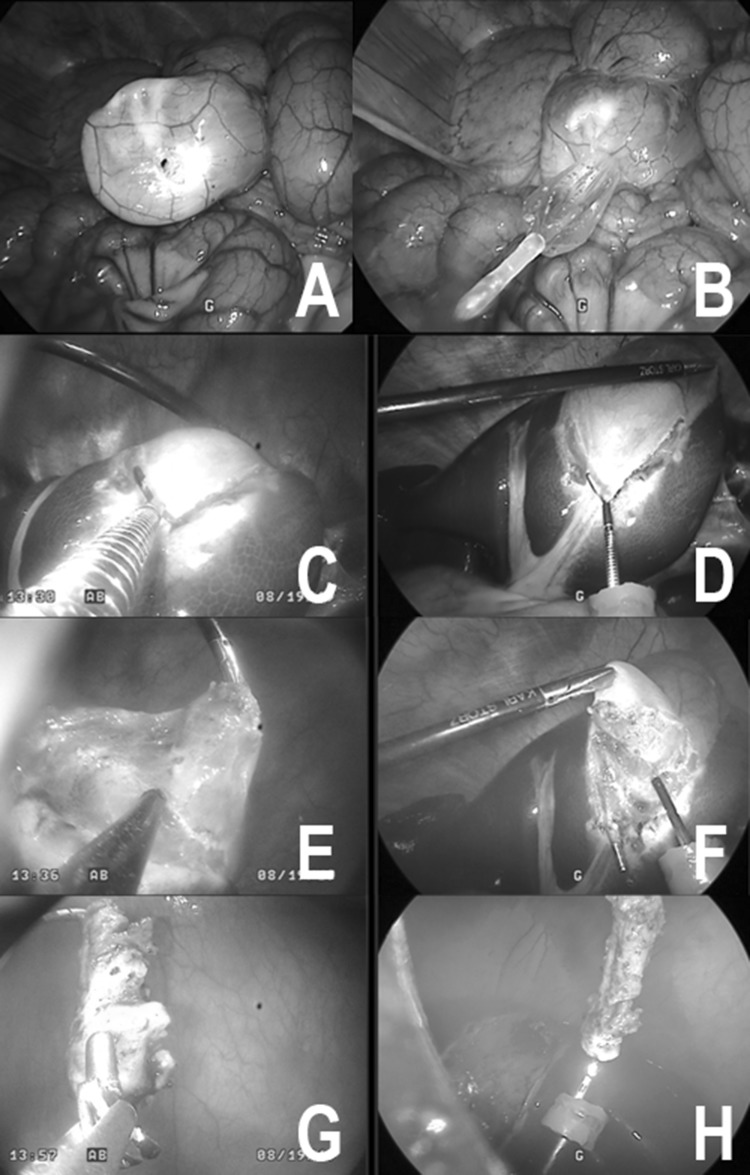
Transluminal MIEO-Port cholecystectomy. Upper left/**A** laparoscopic view showing the docked MIEO-Port from external side after punctual incision of the colon. Upper right/**B** with an inflatable balloon the incision is widened to the diameter of the endoscope. Mid left/**C** Endoscopic view while clipping the cystic duct after dissection. Mid right/**D** corresponding laparoscopic view. Lower left/**E** Dissection of gallbladder from liver bed with repeated saline injections. Lower right/**F** corresponding laparoscopic view. Bottom left/**G**: after dissection the gallbladder is retrieved with the endoscope. Lower right/**H**: laparoscopic view after dissection

The gallbladder was exposed by a rigid laparoscopic manipulator that was inserted transabdominally for this purpose only. The angle of Calot was dissected and the cystic artery and duct were cut after endoscopic clip closure. The dissection of the gallbladder was facilitated by repetitive saline injection for separation from the liver bed. After dissection, the gallbladder was withdrawn via the MIEO-Port which was confirmed stable in its prior position. The procedure took a total of 57:50 min to complete, with 19:33 min from insertion of the scope until exposure of the gallbladder, 35:14 min for the dissection and 2:04 for the retrieval. The port system could easily be released from the entry site; closure of the perforation site was omitted as with a non-survival approach. No complications or malfunctions of the port were registered throughout the experiment; however, stability of the endoscope was limited due to the flexible design of the MIEO-Port (Fig. 4).

### Transluminal MIEO peritoneoscopy with survival

The survival animal study with follow-up over 10 days was focused on intraoperative problems with the MIEO-Port, infectious complications at the entry site and peritoneal cavity as well as on local complications on the docking site. For the survival study, we performed a diagnostic peritoneoscopy over at least 10 min. The penetration technique and access to the peritoneal cavity followed the principles as described previously.

**Fig. 5:**
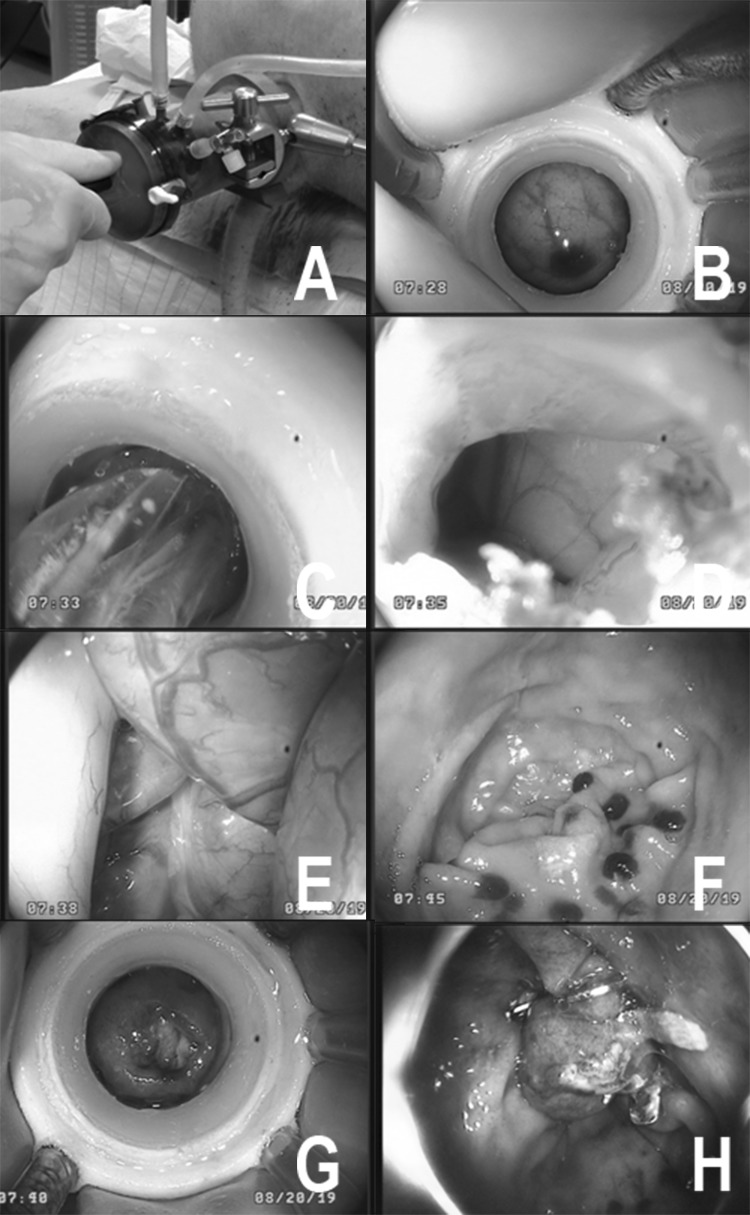
Transluminal MIEO-Port peritoneoscopy. Upper left/**A** external view showing the docked MIEO-Port after intubation of the anus and with the outer cap sealing the natural orifice Upper right/**B** orifice after docking the intestinal wall and cleansing of the entrance site, the endoscope was freed from the overtube. Mid left/**C** Endoscopic balloon dilatation of the perforation site. Mid right/D: view before passing the scope intra-abdominally. Lower left/**E** Endoscopic peritoneoscopy. Lower right/**F** Endoscopic exposure after withdrawal of the endoscope from the abdominal cavity. The MIEO-Port is fixed in place well sealing the entrance from the contaminated intestine. Bottom left/**G** after releasing the MIEO-Port from the intestine small haemorrhages remain that encircle the perforation site. Bottom right/**H** endoscopic view of the penetration site after closure by means of OTS clip application

To summarize, the interventions took a mean of 47:39 min with a mean time from introduction to penetration of 12:43 min and 33:31 min from access to closure. Docking and disinfection worked without problems in four of five animals; in the first animal (No. 1), however, the inner fixation balloon presented an air leak so the endoscope got loose before attaching the port to the intestinal wall. The port was withdrawn and the leaking site repaired. Subsequently, all ports worked fine for docking as did the disinfection and incision of the intestinal wall. In one animal (No. 3), the entry site was chosen too low resulting in a retroperitoneal incision and preparation. After endoscopic manipulations over more than 20 min without accomplishing peritoneoscopy the experiment was finished. In the same animal a dislocation of the port was noticed whilst withdrawal of the endoscope. Closure of the access site was successful in all animals (Fig. 5).

**Fig. 6:**
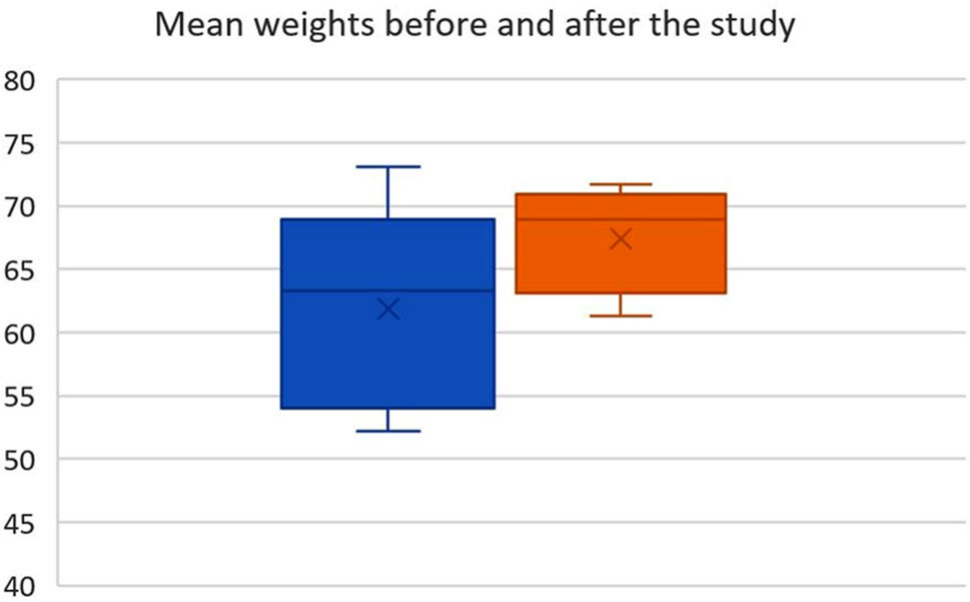
Plot of the mean weights of animals in the survival study. The blue bar indicates the preoperative weight and the orange bar the postoperative weight respectively

All animals showed an uneventful postoperative course without evidence for complications (no signs of pain, peritonitis or infection). Mean weight gain accounted for 5.56 kg including the first animal losing weight of 4.2 kg in 10 days (Fig. 6). For this animal and despite the inconspicuous finding during post-mortem examination, bacterial swabs revealed a bacterial contamination that might have resulted in a subclinical infection. All results and development of weight gain of animals in the survival study are summarized in Table 1.

**Table 1:** Overview on results in the survival animal study

Animal no	1	2	3	4	5
Pre-test weight	73,1	55,8	64,8	63,3	52,2
Procedure time	42:36	32:14	47:04	52:13	64:11
Time until disinfection	10:41	08:05	3:35	21:47	19:26
Time after disinfection	31:14	20:33	42:32	29:48	43:30
Peritoneal access	*Yes*	*Yes*	***No***	*Yes*	*Yes*
Port dislocation	*No*	*No*	***Yes***	*No*	*No*
Local complications	*No*	*No*	*No*	*No*	*No*
Peritoneal complications	*No*	*No*	*No*	*No*	*No*
Bact. contamination abd.*	***Pos***	*Neg*	*Neg*	*Neg*	*Neg*
Bact. contamination local*	***Pos***	*Neg*	*Neg*	**Pos.**^**2**^	**Pos.**^**2**^
Final weight	68,9	61,3	70,1	71,7	65
Weight development	− 4,2	5,5	5,3	8,4	12,8

**Fig. 7:**
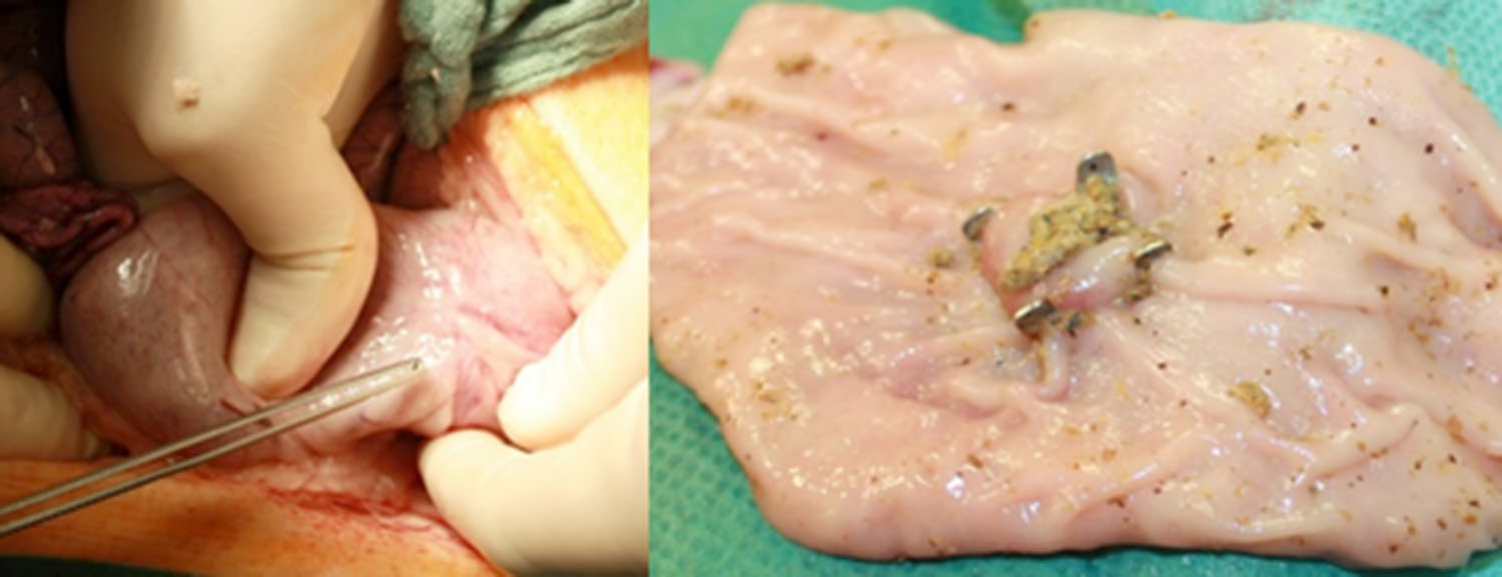
After survival over 10 days and during post-mortem examination, we could not find any infectious or surgical complications. On left sided the external aspect of the completely healed penetration site is indicated, while the luminal aspect is shown on the right

During necropsy, all animals had unsuspicious findings, both for peritoneal or local visual exploration. The bacterial swabs of the peritoneum and the entry site were free of bacterial growth in two of five animals. In animal No. 1 microbiologic testing revealed positive findings for E.coli both abdominally and locally, in animal Nos. 4 and 5 Enterococcus subspecies were identified after enrichment of swab cultures taken from the penetration site. The entry site and surrounding tissue were well healed in all animals with the OTS® clip in situ. There were no side effects or complications to be noticed as a consequence of the docking technique (Fig. 7).

## Discussion

Reducing the interventional trauma by promoting transluminal surgery still is highly attractive, although the hype on this kind of intervention has faded, if not completely disappeared over the last years. Notwithstanding, some studies and meta-analyses assume advantages for transluminal surgery concerning the postoperative pain [15–17] and the cosmetic result while meeting overall complication and success rates of laparoscopic surgery. Still, the evaluation of transluminal techniques continues, e.g. for specimen retraction [18] or for gastroenterostomy [19] indicating a sustained belief in this method. However, human application is still limited and can maybe be attributed to three central aspects or, explicitly, the unavailability of suitable instruments [20, 21], missing indications, and unavailability of a universal and safe port system.

The safe and sterile access to the peritoneal cavity is an absolute requirement for transluminal surgery and for transcolonic surgery in special. According to Kantsevoy [22], different phases of a transluminal procedure have to be considered with the access phase, the intervention phase and the postoperative course being the most relevant. In other words, any access technique for transluminal surgery must prevent from contamination during the penetration of the intestinal wall, must seal the entrance site during the entire intervention, and finally must offer a highly reliable closure technique.

With the MIEO-Port we did not aim only to address all of the aforementioned aspects, but to develop a rather universal port system, that can be used anywhere along the gastrointestinal tract, irrespective if applied via the anus or mouth.

For contamination control, which by far is of higher relevance than universal applicability, two key innovations are featured by the MIEO-Port. First the reversible docking to the intestinal wall by suction that leads to separation and sealing of the penetration site. This principle to the best of our knowledge has not been described before and allowed in prior tests for intestinal attachment of the port with sufficient strength (> 2 N) and with hermetically sealing the docking site. The principle worked reliably well during the ex vivo tests and also during the presented in vivo evaluation; however, it was associated with tiny haemorrhages at the entry site caused by vacuum suction. As we could not exclude post-fixation necrosis or fistulas due to ischemia or mechanical alterations at the docking site, the evaluation of the intestinal wall surrounding the entrance site was of special interest. Following observation over 10 days and after a median fixation time with continuous suction of more than 30 min, fortunately, we could not identify any related complications, nor did we register any remnant of these spots, even after careful exploration. The intestine was healthy and intact without adhesions and even from the inside no scars or alterations were noticed. Accordingly, but limited by the small study size and the short duration of the chosen intervention, we assume the principle of coupling by vacuum suction as reliable.

However, in one case a dislocation of the MIEO-Port had to be observed during withdrawal of the endoscope. We really can not reason this, but assume that the extensive manipulations after retroperitoneal incision might have resulted in forces which exceeded our laboratory specifications. As in any other experiment with peritoneal access, as well as in all ex vivo tests the port system had remained fixed to the intestinal wall we believe this to be the best explanation. To prevent from such adverse events during later clinical application, we propose usage of a sensor controlled suction pump that registers leakage by increased air flow to signal any dislocation or incomplete docking. Nevertheless, further studies and ex vivo tests are warranted to ascertain the required fixation force for transluminal manipulations, which we assumed to be equal or higher than 2 N according to prior tests.

The second innovative feature of the MIEO-Port is the local decontamination by jetting the intestinal surface with a disinfectant, a principle well known from soft tissue infections [23]. Although we did not evaluate on this in a comprehensive comparative setting, we believe, according to the existing ex vivo tests local jetting with use of Octenidine to be superior to the traditional enemas and irrigation methods [11, 13]. In these tests and in comparison with water, Polyiodvidone and Polyhexanide, only the final procedural method was capable to reduce the intestinal bacterial load to an acceptable low level or zero. These results correspond to others, for example Müller, who noticed a remaining and comparatively high contamination rate of the mucosal surface and translocation of bacteria into the abdominal cavity after he had applied Polyiodvidone enemas [24]. We also believe our technique to be better for other reasons. First, it guarantees by principle the direct and local application of the disinfectant at the penetration site. Second, the straight application of the disinfectant via tubes maintains the original concentration of the applied agent, keeping its biochemical effectiveness. And third, the intestinal surface is cleansed under direct visual control, so the disinfection process can be continued until the intestinal surface visually appears clean. Again, we did not comprehensively evaluate on this item; however, we would rate this conclusion as reproducible and logically consistent. Nevertheless, our investigation showed results which did not convince completely as contamination was registered even after careful disinfection. During post-mortem examination in two animals, we could not register any infections, neither by inspection nor by smear examination and microbiologic testing, whereas in another two animals, minor contamination was identified but only locally. It is unclear whether these positive swabs resulted from the intervention itself, from the postoperative healing process, or maybe from a minor contamination prior to the application of the OTS Clip but would rate this minor contamination to be within the acceptable limit. These results need to be separated from the obvious contamination in the first animal, which we assume to have resulted from the unintended detachment of the endoscope. With the loose endoscope, an unrealized contamination of the endoscope shaft and internal surface of the port might have occurred, which was then translocated to the abdomen.

An ideal access technique finally claims for reliable closure of the penetration site. As we were unable to reuse the proven suture technique formerly chosen for the ISSA [9, 25, 26], we had to find an alternative resilient technique, which was the OTSC (Ovesco, Tübingen, Germany). It is well evaluated for closure of intended [27, 28] and unintended [29, 30] perforations and proved reliability in numerous publications. Furthermore, we believe that the OTSC can be modified for integration into the MIEO-Port, for example if imposed to the head component, which would then enable immediate closure of the entrance. Fortunately, the OTSC again worked perfectly well resulting in the intended safe and reliable closure of the penetration site which was supported by guidance by the docking marks.

Despite being developed as a port system for transluminal surgery, we also evaluated the MIEO-Port for controlled endoscopic mucosectomy. Although our test was unable to prove this suitability, we assume in specific cases and with a suitable pathology the port could become enabling as by supporting underwater dissection or by providing a gauge pressure to control bleeding, as it is known from laparoscopy. These applications are only speculative and require further investigations and maybe also further modifications of the port system. Another potential field of use we suppose could be postoperative complications, for example after failed colon resections, where the MIEO-Port could ease necrosectomy and cleansing of the defect and facilitate controlled irrigation. As a fact, with this study we only focused on the main principle of the MIEO-Port and on its usability during transluminal interventions, but not on the development of a new procedure, so further studies are required.

Admittedly, the presented study has several limitations with the small sample size being the most relevant one. The study design is non-comparative and only focuses on transluminal peritoneoscopies and one cholecystectomy, thus on minor surgeries. As we had to observe one dislocation of the port in one animal after extensive manipulation, further studies must prove the reliability of the port system also for longer and more complex interventions. Although evaluated on in prior ex vivo tests, the disinfection principle likewise requires refinement and definition of a standardized protocol. Accordingly, the presented results are only primary and warrant further studies and optimization of the MIEO-Port.

## Electronic supplementary material

Below is the link to the electronic supplementary material.

Supplementary file1 (WMV 68153 kb)
